# Novel 4-Arm Poly(Ethylene Glycol)-Block-Poly(Anhydride-Esters) Amphiphilic Copolymer Micelles Loading Curcumin: Preparation, Characterization, and In Vitro Evaluation

**DOI:** 10.1155/2013/507103

**Published:** 2013-07-08

**Authors:** Li Lv, Yuanyuan Shen, Min Li, Xiaofen Xu, Mingna Li, Shengrong Guo, Shengtang Huang

**Affiliations:** ^1^School of Pharmacy, Shanghai Jiao Tong University, Shanghai 200240, China; ^2^School of Pharmacy, Hubei University of Science and Technology, Xianning 437100, China

## Abstract

A novel 4-arm poly(ethylene glycol)-block-poly(anhydride-esters) amphiphilic copolymer (4-arm PEG-b-PAE) was synthesized by esterization of 4-arm poly(ethylene glycol) and poly(anhydride-esters) which was obtained by melt polycondensation of **α**-, **ω**-acetic anhydride terminated poly(L-lactic acid). The obtained 4-arm PEG-b-PAE was characterized by ^1^H-NMR and gel permeation chromatography. The critical micelle concentration of 4-arm PEG-b-PAE was 2.38 **μ**g/mL. The curcumin-loaded 4-arm PEG-b-PAE micelles were prepared by a solid dispersion method and the drug loading content and encapsulation efficiency of the micelles were 7.0% and 85.2%, respectively. The curcumin-loaded micelles were spherical with a hydrodynamic diameter of 151.9 nm. Curcumin was encapsulated within 4-arm PEG-b-PAE micelles amorphously and released from the micelles, faster in pH 5.0 than pH 7.4, presenting one biphasic drug release pattern with rapid release at the initial stage and slow release later. The hemolysis rate of the curcumin-loaded 4-arm PEG-b-PAE micelles was 3.18%, which was below 5%. The IC_50_ value of the curcumin-loaded micelles against Hela cells was 10.21 **μ**g/mL, lower than the one of free curcumin (25.90 **μ**g/mL). The cellular uptake of the curcumin-loaded micelles in Hela cell increased in a time-dependent manner. The curcumin-loaded micelles could induce G_2_/M phase cell cycle arrest and apoptosis of Hela cells.

## 1. Introduction

Biodegradable polyanhydrides and polyesters for pharmaceutical applications have received considerable interests in recent decades [1–5]. Polyanhydrides are a particularly promising class of polymers for drug delivery, due to their chemical properties and biocompatibility [6, 7]. The surface-eroding properties of polyanhydrides in aqueous medium make them desirable for controlled drug release [8, 9]. Polyanhydride-based wafers that deliver carmustine for the treatment of brain cancer have been approved by the FDA [10]. Polyesters such as poly(lactic acid), poly(D, L-lactic-co-glycolic acid), and poly(*ε*-caprolactone) have been commonly used to form micelles to deliver a variety of drugs [11]. Recently, poly(anhydride-esters) copolymers have been synthesized, in order to combine advantages of polyanhydrides and polyesters [12–14]. These copolymers possess both the degradation and surface erosion properties of individual polyesters and polyanhydrides and may, therefore, provide extended advantages compared to either polyanhydrides or polyesters alone.

Amphiphilic block copolymers have been used extensively in pharmaceutical applications ranging from sustained-release technology to gene delivery [15]. In aqueous solution, they can form micelle structures with a hydrophobic inner core and hydrophilic outer shell in aqueous media at or above the critical micelle concentration (CMC). The polymeric micelles have unique characteristics, such as nanosize, and good thermodynamic stability in physiological conditions [16]. Upon the CMC, the hydrophobic core regions serve as reservoirs for drugs that have a poor water solubility. Poly(ethylene glycol) (PEG) is often used to build hydrophilic blocks of micelle-forming copolymers, because it is biocompatible, highly soluble, and hydrated in water [16]. Amphiphilic block copolymer composed of PEG and polyanhydride has been prepared by melt copolymerization. By regulating the PEG content, polyanhydrides with adjustable surface erosion rate can be obtained, which are more suitable for a drug-delivery device [17, 18]. In this study, it was expected that a novel 4-arm poly(ethylene glycol)-b-poly(anhydride-esters) amphiphilic copolymers (4-arm PEG-b-PAE) could be obtained by esterization of 4-arm poly(ethylene glycol) and poly(anhydride-esters) which was obtained by melt polycondensation of *α*-, *ω*-acetic anhydride terminated poly(L-lactic acid).

Curcumin, a natural polyphenolic compound isolated from the rhizome of the perennial herb curcuma longa [19–21], has been receiving considerable attention because of its putative cancer prevention and anticancer activities. It was reported that curcumin could interact with many cellular targets such as nuclear factor-kappa B (NF-*κ*B) and transcription factor activator protein-1 (AP-1) [22–24]. Curcumin has antiproliferating effect in some cancer cell lines of human origin [25]. Attractively, clinical trials have proved that curcumin is very safe for humans even at a high dose of 12 g per day [26, 27]. In this study, curcumin was used as a model drug to evaluate the possibility of the 4-arm PEG-b-PAE as a nanocarrier. The 4-arm PEG-b-PAE was characterized by ^1^H-NMR and gel permeation chromatography. The curcumin-loaded 4-arm PEG-b-PAE micelles were prepared by a solid dispersion method, and then the characteristics of the cucumin-loaded micelles (e.g., size, *ζ*-potential, encapsulation efficiency, and drug loading content) were investigated. The in vitro released behavior of curcumin was studied in phosphate buffer solution (PBS, pH 7.4 or pH 5.0) and the stability and hemolysis of the micelles were evaluated. In vitro cytotoxicity, cellular uptake, retardation of cell cycle, and induction of apoptosis of micelles to Hela cells were also reported.

## 2. Materials and Methods

### 2.1. Materials

L-Lactide, diethylene glycol, Sn (II) octoate, succinic anhydride, acetic anhydride, and 4-arm poly(ethylene glycol) (Mn = 20000) were obtained from Aladdin chemistry Co., Ltd. (Shanghai, China). Curcumin was purchased from Alfa-Aesar. Dulbecco's modified eagle's medium was purchased from Invitrogen Corporation (Grand Island, USA). Penicillin-streptomycin, fetal bovine serum (FBS), 0.25% (w/v) trypsine, 0.03% (w/v) EDTA solution, phosphate buffer solution (PBS), and propidium iodide (PI) were purchased from Solarbio Science & Technology (Beijing, China). RNase A and Annexin V-FITC/PI were purchased from Beyotime (Suzhou, China).

### 2.2. Synthesis of Copolymers

**Figure 1:**
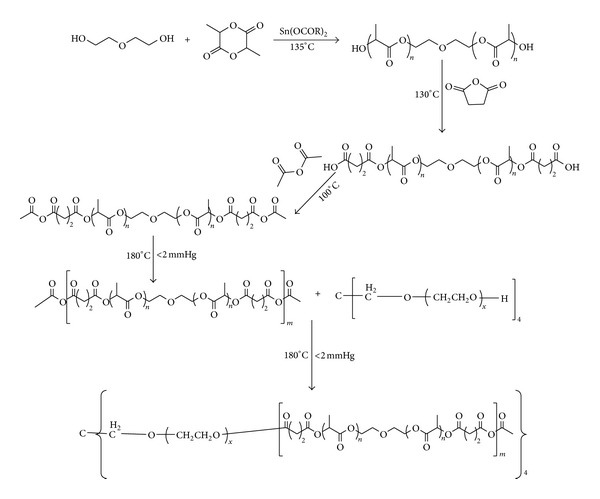
Schematic of synthesis of the 4-arm PEG-b-PAE.

4-arm PEG-b-poly(anhydride-esters) was synthesized by melt polycondensation as shown in Figure 1. Under nitrogen protection, Sn (II) octanoate (100 mg in 1 mL of toluene) was added to the mixture of 3.6 g L-Lactide and 0.87 mL of diethylene glycol. The reaction mixture was heated to 135°C for 25 minutes. The obtained polymer was dissolved in dichloromethane (DCM) and precipitated in ethanol/hexane (2 : 8 v/v) mixture. After drying in vacuum for 48 h, 0.8 g succinic anhydride was added to the polymer and the mixture was heated to 130°C for 8 hours. The obtained polymer was dissolved in DCM. 50 mL of water was introduced and the solution was stirred for 1 hour. The water was separated by a separatory funnel. The polymer was recovered from precipitation in cold diethyl ether and dried under vacuum. After drying the polymer was heated with 60 mL acetic anhydride to 100°C for 8 hours; then the mixture was applied vacuum to remove the excess of acetic anhydride. Once all the unreacted acetic anhydride was removed under vacuum, the temperature was increased to 180°C for 6 hours. The poly L-lactic acid based poly(anhydride-esters) was recovered from precipitation of the polymer in cold diethyl ether and dried under vacuum.

 4-arm PEG (0.0004 mol) and poly L-lactic acid based poly(anhydride-esters) (0.0016 mol) were added to the reactor and then were heated to 180°C, under vacuum for 12 hours. After polycondensation the polymer was dissolved in DMC and precipitated in cold petroleum benzin and dried under vacuum.

### 2.3. Characterization of Copolymers

#### 2.3.1. ^1^H-NMR

^1^H-NMR spectra were measured on a Varian Mercury plus 400 spectrometer with CDCl_3_ as solvent. The tetramethylsilane (TMS) was used as an internal standard.

#### 2.3.2. Gel Permeation Chromatography (GPC)

GPC measurements were performed at 40°C on a Waters HPLC system equipped with a model 1525 binary HPLC pump, a model 2414 refractive index detector and a series of 110 Styragel columns (HR_3_ and HR_4_). Tetrahydrofuran was used as an eluent at a flow rate of 1.0 mL/min. The molecular weights were calculated by using a calibration curve which was constructed using polystyrene as standard.

#### 2.3.3. Critical Micelle Concentration (CMC)

A steady-state pyrene fluorescence method was used to determine the CMC of the copolymer according to the method described previously [28]. Briefly, pyrene was dissolved in acetone and added to 5 mL volumetric flasks to provide a concentration of 6 × 10^−7^ M in the final solutions. Acetone was then evaporated and replaced with aqueous polymeric micelle solutions with concentrations ranging from 0.1 to 100 *μ*g/mL. Fluorescence spectra were recorded on a Hitachi F-7000 spectrophotometer. The scans were performed at medium speed (240 nm/min) and at a PMT detector voltage of 350 V. The emission wavelength was carried out at 395 nm and the excitation spectra were recorded ranging from 300 to 350 nm with both bandwidths set at 5 nm. A CMC value was determined from the ratios of pyrene intensities at 337 (*I*_337_) and 335 (*I*_335_) nm and calculated from the intersection of two tangent plots of *I*_337_/*I*_335_ versus log concentrations of copolymers.

### 2.4. Preparation of Micelles

The curcumin-loaded micelles were prepared by a solid dispersion method. Briefly curcumin (5 mg) and 4-arm PEG-b-PAE (50 mg) were dissolved in 10 mL of dichlormethane by sonication. The organic solvent was evaporated on a rotary evaporator under reduced pressure at 40°C to obtain a homogenous coevaporation curcumin/copolymer matrix. The resulting matrix was hydrated by adding 20 mL of water and then sonicated at 200 w for 5 min; the power was pulsed for 5 s every 30 s to minimize increases in temperature. The formed suspension was centrifuged at 4000 rpm for 20 min to remove the aggregated particles and unencapsulated free curcumin, and then filtered through a 0.45 *μ*m filter (Agela Technologies Inc., China), followed by lyophilization for 3 days to get the powdered form of micelles. Empty 4-arm PEG-b-PAE micelles were prepared by the same way.

### 2.5. Characterization of Polymeric Micelles

#### 2.5.1. Particle Size, Size Distribution, and *ζ*-Potential

The average particle size and polydispersity (PDI) of micelles were determined by dynamic light scattering (DLS) using a Malvern Instrument zeta size nano-s at a detection angle of 173°. *ζ*-potential was measured using the BC Haven instruments corporation 90 plus particle size analyzer. The samples were dispersed in distilled water at 25°C and diluted to a suitable density. 

#### 2.5.2. Transmission Electron Microscopy (TEM) Morphology

The morphological characteristics of polymeric micelles were examined by transmission electron microscopy (TEM, Tecnai G_2_ spirit Biotwin). The curcumin-loaded micelles were diluted with distilled water and placed on a copper grid covered with nitrocellulose.

#### 2.5.3. Evaluation of the Drug Encapsulation Efficiency and Drug Loading Content

The amount of encapsulated curcumin in the micelles and the drug loading content were evaluated by direct method. Briefly, 10 mL of acetonitrile was added to 2 mg of the freeze-dried micelles powder in order to disrupt the micelles structure. The solution was dried under nitrogen, then dissolved in mobile phase, centrifuged at a rate of 10000 rpm for 10 minutes, and filtered through 0.45 *μ*m filter to obtain a clear solution; then the sample solution was determined at 425 nm by using a Shimadzu HPLC system, equipped with an LC-10ADvp pump, an SPD-17310 Avp UV-vis detector and a Diamonsil C_18_ reversed phase column 174 (4.6 mm × 250 mm, 5 *μ*m). The mobile phase was composed of an acetonitrile/3% acetic acid (75/25, v/v) at a temperature of 30°C and a flow rate of 1.0 mL/min. The encapsulation efficiency and drug loading content were calculated using (1) as follows:

(1) Encapsulation  efficiency(%) =weight  of  curcumin  in  micellesweight  of  feeding  curcumin×100%,Drug  loading  content(%) =weight  of  curcumin  in  micellesweight  of  micelles×100%.

#### 2.5.4. Crystallographic Study

Crystallographic assay was performed on curcumin powders, empty 4-arm-PEG-PAE micelles, and the curcumin-loaded 4-arm PEG-b-PAE micelles free-dried powers by a Rigaku 195 D/max 2200/PC diffractometer using a Cu-K*α* radiation source (40 kV, 20 mA). The samples were scanned over a 2*θ* range of 5–45° at a rate of 5°/min.

### 2.6. In Vitro Release Study

In vitro release behavior of curcumin from the 4-arm PEG-b-PAE micelles was developed by dialysis method [29]. Briefly, the curcumin-loaded 4-arm PEG-b-PAE micelles (10 mg) were dispersed in 3 mL PBS (0.01 M, pH 7.4 or pH 5.0) containing 1% v/v of Tween 80 (to maintain a sink condition) and then placed in a dialysis tube (Snakeskin, Pierce, USA) with a molecular weight cut-off of 3500 Da. The dialysis tube was suspended in 12 mL of release medium and placed in a shaking water bath at 37°C with a shaking speed of 120 rpm. At predetermined time points, the release medium was completely drawn and replaced with fresh incubation medium. The amount of curcumin in the release medium was determined by HPLC.

### 2.7. Hemolysis by the Curcumin-Loaded Micelles

Briefly fresh blood from Sprague-Dawley rat was collected in heparinized tubes and washed three times with 0.9% NaCl by centrifugation at 2800 rpm for 5 min and suspended in 0.9% NaCl solution (2% v/v). Erythrocytes suspension (0.1 mL) was added to 0.9 mL of the curcumin-loaded micelles (100 *μ*g/mL), 0.9% NaCl solusion (negative control group with 0% hemolysis) and distilled water (positive control group with 100%), respectively, then incubated for 2 hours in a shaker incubator at 37°C. After centrifugation at 3000 rpm for 10 min, the absorbance of the supernatant was determined at 540 nm. The degree of hemolysis was determined by the following equation:

(2) $$\begin{array}{c} \text{Hem}\left(\%\right)=\frac{\text{ABS}-{\text{ABS}}_{0}}{{\text{ABS}}_{100}-{\text{ABS}}_{0}}\times 100, \end{array}$$

where ABS_100_ and ABS_0_ are the absorbances of the solution at 100% and 0% hemolysis, respectively. 

### 2.8. Characteristics of Micelles Uptake

The characteristics of micelles cellular uptake were studied by flow cytometry. Briefly, Hela cells (3.0 × 10^5^ cells in 2 mL medium) were seeded in 6-well plates and incubated overnight. The medium was removed, and the cells were washed twice with PBS and incubated in serum-free medium for different times with the curcumin-loaded 4-arm PEG-b-PAE micelles (curcumin concentration was 10 *μ*g/mL) for 24 h at 37°C. Cells treated with only medium were used as control. After incubation, the cells were washed three times with ice-cold PBS, and then cells were collected by centrifugation and resuspended in 0.5 mL PBS. The amount of uptake was analyzed by a flow cytometry (BD LSRFortessa, Becton Dickinson), after excitation with 405 nm argon lasers and detection with a 515–545 band-pass filter.

### 2.9. In Vitro Cytotoxicity

The in vitro antitumor activity of the curcumin-loaded micelles and free curcumin was determined by MTT assay. Briefly, Hela cells (obtained from Shanghai Institute of Cell Biology, Chinese Academy of Sciences) in their logarithmic growth were seeded in 96-well plates at a density of 5000 cells per well in a final volume of 200 *μ*L medium and allowed to adhere overnight. The culture medium was carefully replaced with 200 *μ*L of media containing DMSO-dissolved curcumin (final DMSO concentration ≤ 0.1%) or the curcumin-loaded micelles at curcumin concentrations ranging from 1 to 40 *μ*g/mL. Cells were incubated with treatments for 72 hours. Then the viability of the cells was determined using MTT assay. The cells were incubated with 200 *μ*L medium containing 0.5 mg/mL MTT for another 4 hours, allowing the visible cells to transform the yellow MTT into dark-blue formazan crystals, which were dissolved in 200 *μ*L of DMSO. The absorbance values were measured at 570 nm by an ELISA plate reader (Varioskan Flash). Hela cell viability is defined as the percent live cells compared with untreated controls. The percentage of Hela cell viability was calculated as follows:

(3) $$\begin{array}{c} \text{Hela}\;\text{cell}\;\text{viability}\;\%=\frac{{\text{A}570}_{\text{sample}}}{{\text{A}570}_{\text{control}}}\times 100\%. \end{array}$$

In this assay, all the experiments were done with ten parallel samples. Untreated cells, culture medium containing 0.1% DMSO, and culture medium containing blank micelles were also tested as controls.

### 2.10. Flow Cytometric (FCM) Analysis of Cell Cycle Distribution

Flow cytometry provides information on cell cycle phases and is sensitive to apoptosis based on bivariate analysis of DNA content. For the analysis of cell cycle distribution, Hela cells were seeded at a density of 1.5 × 10^5^ cells/mL (2 mL/well) in 6-well plates 24 hours before the experiment. The cells were exposed to either free curcumin or the curcumin-loaded micelles at the curcumin concentration of 50 *μ*g/mL in culture medium and incubated for 72 h in CO_2_ incubator at 37°C. Cells treated with only medium were used as controls. After incubation, the cells were washed with cold PBS for three times. The cells were collected by centrifugation and fixed with 70% precooled alcohol overnight at 4°C. Before measurements, the cells were washed to eliminate alcohol and then incubated with RNase A (0.1 mg/mL) for 30 min at 37°C, then stained with PI solution (0.1 mg/mL) for 30 min in the dark. After that the distribution of different DNA contents was analyzed by a flow cytometer (FACS Calibur, BD, USA). For each cell population, at least 10000 cells were examined.

### 2.11. Annexin V-FITC/PI Double Staining

AnnexinV-FITC/PI was used to stain the cells and the percentage of cell apoptosis was determined by flow cytometer. Hela cells were seeded at a density of 1.5 × 10^5^ cells/mL (2 mL/well) in 6-well plates 24 hours before the experiment. The cells were exposed to either free curcumin or the curcumin-loaded micelles at the curcumin concentration of 50 *μ*g/mL in culture medium and incubated for 24 h in CO_2_ incubator at 37°C. Cells treated with only medium were used as controls. At the end of the treatment period, the cells were harvested and washed twice with cold PBS. Then 1 × 10^5^ cells were collected by centrifugation and gently resuspended in 200 *μ*L binding buffer; thereafter 5 *μ*L Annexin V-FITC and 10 *μ*L PI (200 *μ*g/mL) were added and incubated the cells in the dark for 15 min. Finally, 300 *μ*L binding buffer was added into the samples before they were analyzed by a flow cytometer (FACS Calibur, BD, USA).

### 2.12. Statistical Analyses

All the experimental data were expressed as means ± standard deviation (SD). Statistical analyses were performed using a Student's *t*-tests. The significance of differences between groups was considered as *P* < 0.05 and very significant for *P* < 0.01.

## 3. Result and Discussion

### 3.1. Synthesis and Characterization of Copolymers

The 4-arm PEG-b-PAE was synthesized via a five-step synthetic route as illustrated in Figure 1. L-lactide was polymerized in the presence of Sn (II) octoate and diethylene glycol, yielding hydroxyl-terminated poly(L-lactic acid). The hydroxyl-terminated poly(L-lactic acid) was converted to carboxyl-terminated poly(L-lactic acid) by reaction with succinic anhydride. And then the carboxyl-terminated poly(L-lactic acid) reacted with an excess of acetic anhydride, followed by melt polycondensation to obtain the PAE. The 4-arm PEG-b-PAE was prepared by esterization of the PAE and 4-arm-PEG_20k_.

**Figure 2:**
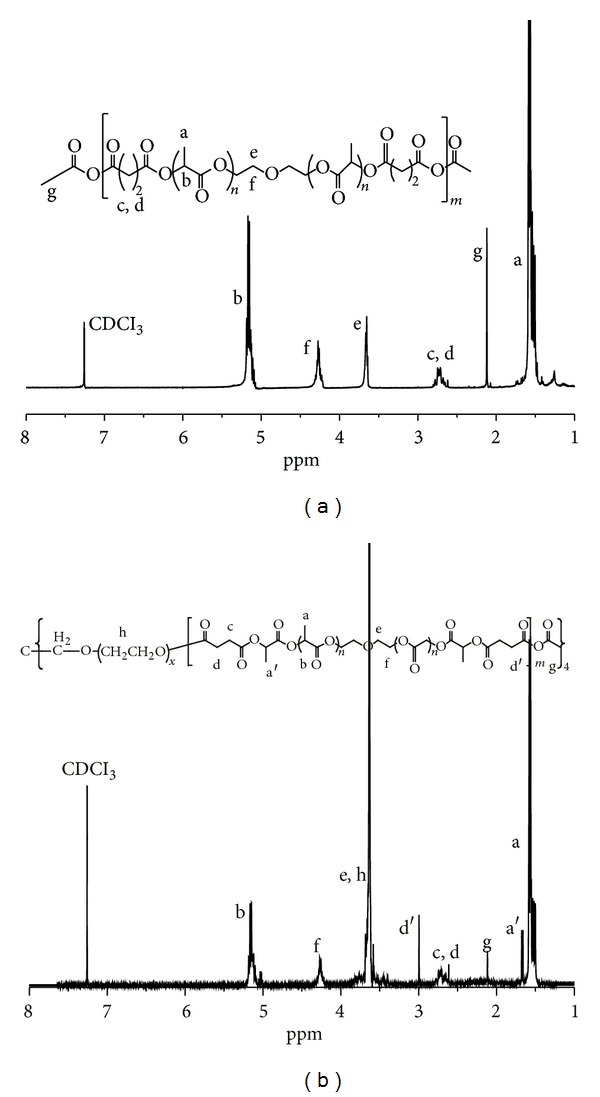
^1^H-NMR spectra of (a) PAE and (b) 4-arm PEG-b-PAE.

The obtained polymers were characterized by ^1^H-NMR and GPC. Figure 2 shows the ^1^H-NMR spectra of the PAE and the 4-arm PEG-b-PAE. It could be found from Figure 2(a) that the peaks at *δ*1.56–1.60, 2.23, 2.75–2.79, 3.64–3.66, 4.26–4.30, and 5.13–5.18 ppm were attributed to the protons of a, g, c-d, e, f and b of the PAE structure, respectively. In Figure 2(b), the sharp peak at 3.57–3.88 was attributed to methylene protons of the –CH_2_CH_2_O– in the 4-arm-PEG block of the 4-arm PEG-b-PAE. Peaks at 1.50–1.58, 1.66–1.68, 2.10, 2.62–2.78, 2.99, 4.25–4.27, and 5.02–5.18 ppm were attributed to the corresponding protons in the PAE block of the 4-arm PEG-b-PAE, respectively. According to the peak areas of Hg, He in Figure 2(a), the molecular weight of the PAE was calculated. The number of repeat units of anhydride was calculated according to ^1^H-NMR, and the value of the PAE is about 9.Table 1 shows the chemical compositions and molecular weights of the synthesized polymers. High polydispersity of the product of coupling looks apparent from the PDI of the final polymer sample which increases from 1.58 for PAE copolymer to 2.65 for 4-arm PEG-b-PAE. It should be noted that the polydispersity was determined using the linear polystyrene molecular weight standards for calibration in this study, which is not very suitable for nonlinear polymers, such as 4-arm PEG-B-PAE. Thus, it is difficult to compare the polydispersity data of PAE and 4-arm PEG-B-PAE accurately.

### 3.2. Critical Micelle Concentration

**Figure 3:**
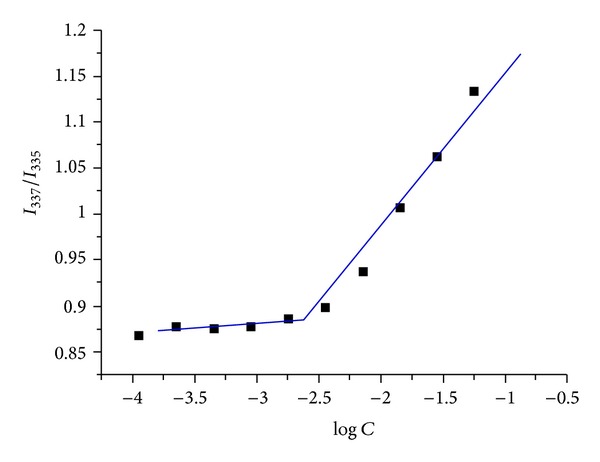
Plots of intensity ratios *I*_337_/*I*_335_ from pyrene excitation spectra versus log concentrations of the 4-arm PEG-b-PAE.

Fluorescence spectroscopy was used to determine the CMC of the copolymer. Pyrene was used as probe to determine the association and micellization of the copolymer in solution. Pyrene had a very low solubility in water and a strong hydrophobic character, so it preferentially solubilized itself in the hydrophobic region of micelles. The process resulted in a great change of the fluorescence intensity. A shift of the peak of the excitation spectra could be observed with the changing concentration of micelles. The maximum peak for pyrene in water was about 335 nm; the peak shifted to 337 nm in the solution of copolymer. So the CMC could be determined by taking a midpoint of the copolymer concentration at which point the ratio of *I*_337_/*I*_335_ was varied. As shown in Figure 3, *I*_337_/*I*_335_ was low and kept constant at low copolymer concentrations and sharply increased as the copolymer concentration increased after the copolymer concentration reached a threshold that is CMC. The CMC of the 4-arm PEG-b-PAE was 2.38 *μ*g/mL, which was significantly lower than that of low molecular weight surfactants in water [30] and implied the good thermodynamic stability of the 4-arm PEG-b-PAE micelles. So, it can be concluded that the 4-arm PEG-b-PAE can form stable micelles structures at its low concentration after dilution with a large volume of body fluids.

### 3.3. Preparation, Characterization, and Stability of Polymeric Micelles

**Figure 4:**
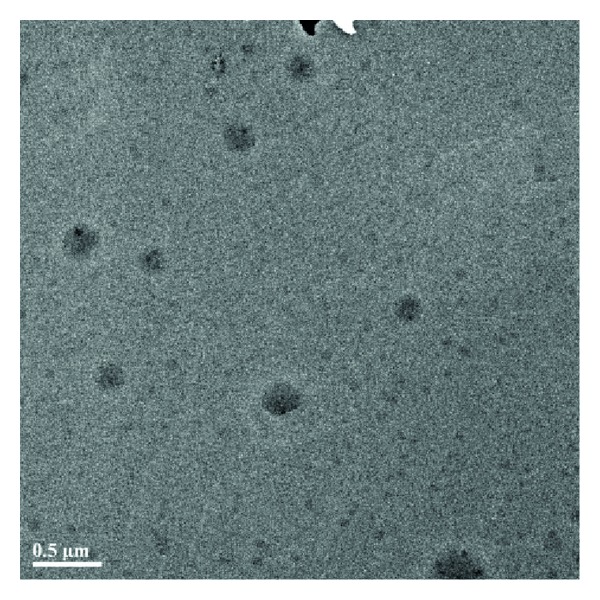
TEM image of the curcumin-loaded 4-arm PEG-b-PAE micelles.

The 4-arm PEG-b-PAE could readily produce self-assembled micelles in aqueous solution because of its amphiphilic property. The PAE part could constitute an internal hydrophobic core, while 4-arm-PEG could provide a hydrophilic outer shell of the micelles. The curcumin-loaded micelles were prepared by a solid dispersion method. The formation of micelles was confirmed by measuring particle size, size distribution, *ζ*-potential, and morphology. The hydrodynamic diameter and zeta potential values were measured by DLS and are shown in Table 2. The hydrodynamic diameter of the particles is expressed in Z-average (nm) and the charge as *ζ*-potential (mV). It shows that the hydrodynamic diameters of empty micelles and the drug loaded micelles are less than 200 nm, with an acceptably good polydispersity index (PDI < 0.09). The zeta potential value changes a little after loading curcumin into the micelles. TEM photograph showed the curcumin-loaded micelles were spherical (Figure 4). Because of these properties, it is assumed that the curcumin-loaded micelles can effectively accumulate in the tumor region via the permeability and retention effect and exhibit reduced uptake by reticuloendothelial system [31].

**Figure 5:**
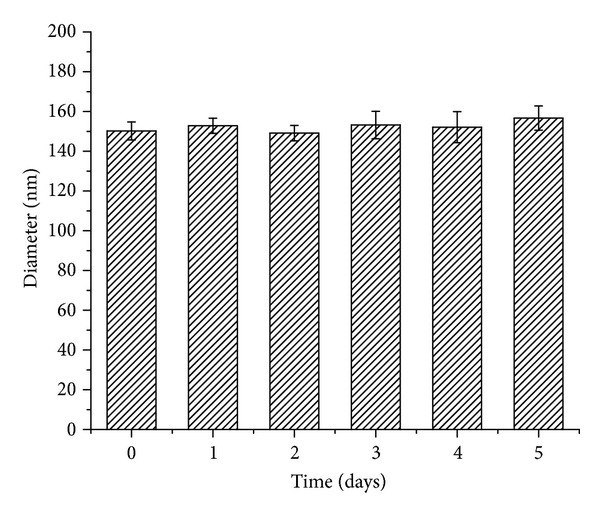
Hydrodynamic diameters of the curcumin-loaded 4-arm PEG-b-PAE micelles versus time (pH 7.4 PBS, 4°C).

The physical stability of micelles in PBS (pH 7.4) at 4°C for 5 days was assessed. No phase separation and no significant change in hydrodynamic diameter of micelles were observed (Figure 5). These results demonstrated the micelles were physically stable during the study. Owing to the high surface/volume ratio, micelles always tend to form aggregates. However, due to electrostatic interactions or/and stereospecific blockade, a stable micelles suspension can also be obtained. The zeta potential of the curcumin-loaded micelles is approximately −10 mv, indicating that micelles presented negative surface charge. Meanwhile, 4-arm PEG-b-PAE copolymer is amphiphilic and consisted of hydrophilic 4-arm PEG blocks and hydrophobic PAE blocks. In aqueous solution, the relative difference in hydrophobicity between the 4-arm PEG and the PAE blocks allows the formation of self-assembled micelles. Therefore, the stability of the curcumin-loaded micelles might be mainly contributed to the stereospecific blockade formed by the hydrophilic 4-arm PEG chain located at the surface of the curcumin-loaded micelles and the electrostatic interactions of micelles.

Drug encapsulation efficiency and drug loading content are important factors for drug delivery systems. An HPLC assay was used to determine the drug concentration in the micelles and then calculated the drug loading content and encapsulation efficiency. As it is seen from Table 2, the drug loading content of the 4-arm PEG-b-PAE micelles was 7.0 ± 0.2% and the encapsulation efficiency of the micelle was 85.2 ± 1.2%, which achieve high drug encapsulation efficiency. 

**Figure 6:**
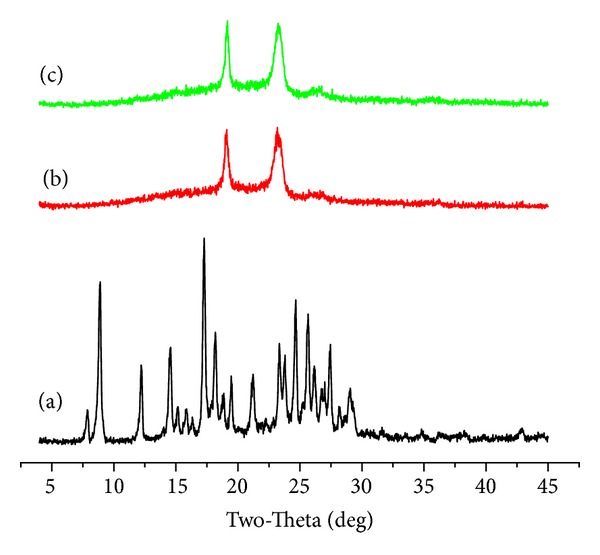
X-ray diffraction spectra of (a) curcumin, (b) empty 4-arm PEG-b-PAE micelles, (c) the curcumin-loaded 4-arm PEG-b-PAE micelles.

To examine the crystallinity of micelle-encapsulated curcumin, XRD analysis was performed on the curcumin-loaded micelles. Figure 6 presents the XRD spectra of the curcumin powders, empty 4-arm PEG-b-PAE micelles, and the curcumin-loaded 4-arm PEG-b-PAE micelles. It could be found that the characteristic diffraction peaks of the curcumin were absent in the spectrum of the curcumin-loaded micelles, which might suggest the curcumin was encapsulated within the micelles amorphously.

### 3.4. In Vitro Curcumin Release

**Figure 7:**
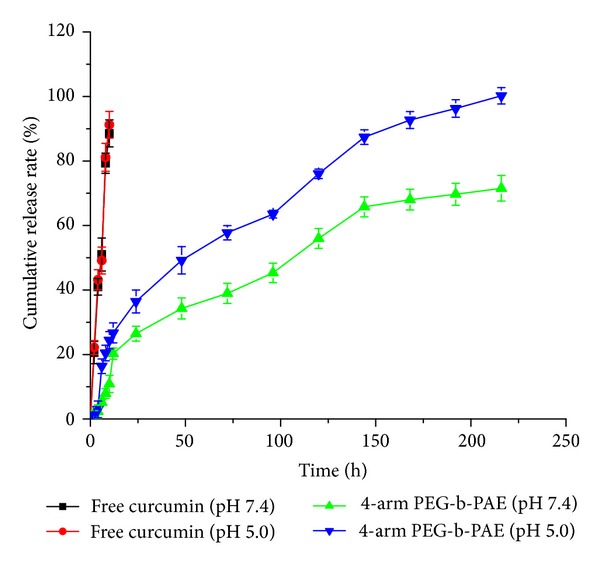
Drug release profiles of the curcumin-loaded 4-arm PEG-b-PAE micelles.

Figure 7 shows the cumulative release profiles of curcumin from the curcumin-loaded micelles under different pH conditions (pH 5.0 and 7.4) at 37°C. Curcumin release from the micelles was monitored for 9 days. Sustained drug release was observed and it was dependent on the pH of the release medium. The cumulative release of curcumin from the curcumin-loaded micelles could reach up to 99.5% after 9 days at pH 5.0, higher than that at pH 7.4, which was 70%. Curcumin released from the micelles consisted of two phases. An initial fast release for about 12 h was followed by a sustained released of drug from the micelles. In Phase 1, the drug adsorbed on the surface of the micelles was separated from the carrier material into the aqueous phase, which contributed to the burst release stage. 

### 3.5. Hemolysis of Micelles

The hemolysis rate of the curcumin-loaded 4-arm PEG-b-PAE micelles was 3.18 ± 1.27%, which was below 5%, indicating that the micelles will not lead to severe hemolysis, according to ISO 10993-4:2002. Therefore, it is suggested that the curcumin-loaded micelles have no destructive effect on erythrocyte.

### 3.6. Cellular Uptake of the Curcumin-Loaded Micelles

**Figure 8:**
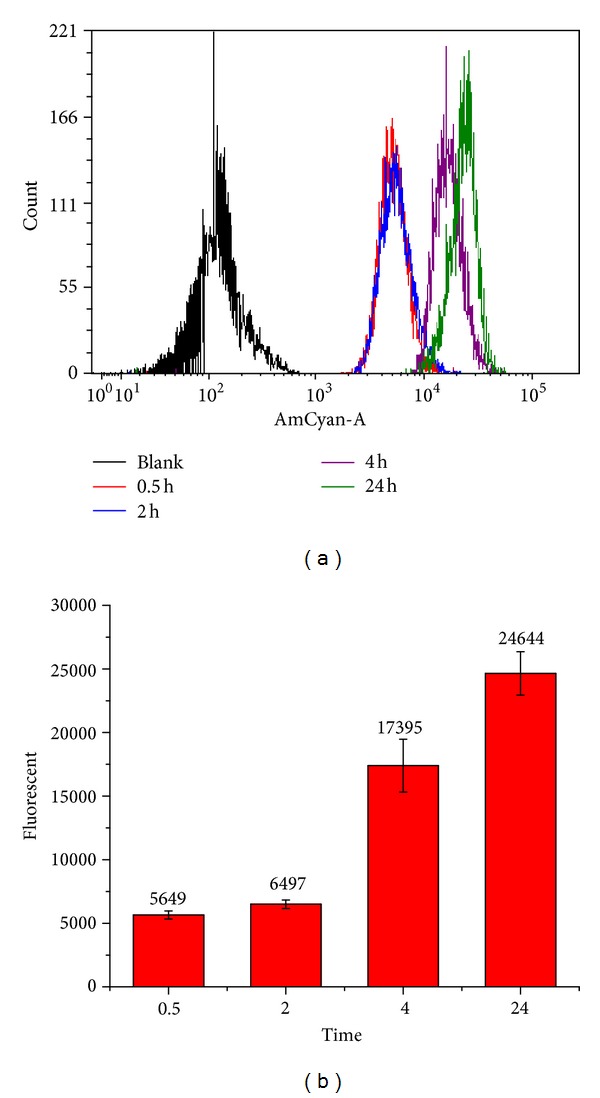
Cellular uptake of the curcumin-loaded 4-arm PEG-b-PAE micelles by Hela cells at 37°C using flow cytometer. (a) Flow histograms of cellular uptake. (b) Mean fluorescence intensity of Hela cells incubated with the curcumin-loaded 4-arm PEG-b-PAE micelles.

Cellular uptake of the curcumin-loaded micelles in Hela cell was investigated by flow cytometer. To investigate the effect of time on the micelles uptake by Hela cells, cells were incubated with the micelles for various periods and the mean fluorescence intensity of cells was analyzed. The results were shown in Figure 8. The cells untreated with the micelles were used as negative control to direct autofluorescence. It can be found that the histograms of 0.5 and 2 hours move to right and the histograms of 4 and 24 hours move much more to right, especially the histogram of 24 hours, comparing with the histogram of blank. The mean fluorescence intensity of cells after incubation of 0.5, 2, 4, 24 hours was 5649, 6497, 17395, and 24644, respectively. These results demonstrate that the cellular uptake of the curcumin-loaded micelles in Hela cell increased in a time-dependent manner.

### 3.7. In Vitro Cytotoxicity

**Figure 9:**
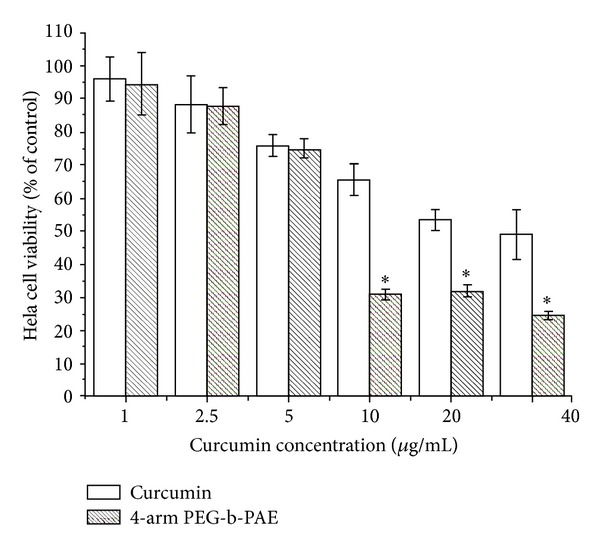
Cytotoxicity of the curcumin-loaded 4-arm PEG-b-PAE micelles against Hela cells. Incubation time was 72 h, data were presented as mean ± SD (*n* = 10). *Significant difference between the curcumin-loaded 4-arm PEG-b-PAE micelles and free curcumin (*P* < 0.05).

As shown in Figure 9, the cytotoxicity of both the curcumin-loaded micelles and free curcumin to Hela cells at 72 hours showed significant concentration dependence. Half-maximal inhibitory concentrations (IC_50_) of the curcumin-loaded micelles and free curcumin to Hela cells were 10.21 *μ*g/mL and 25.90 *μ*g/mL, respectively. From these results, it can be observed that the curcumin-loaded micelles have better efficiency than free curcumin. In addition, the toxicity of the 4-arm PEG-b-PAE used to delivery curcumin was tested. No cytotoxicities at the test range concentrations of the 4-arm PEG-b-PAE were observed at 72 h (data not shown).

### 3.8. The Curcumin-Loaded Micelles Induced Cell Cycle Arrest in Hela Cells

**Figure 10:**
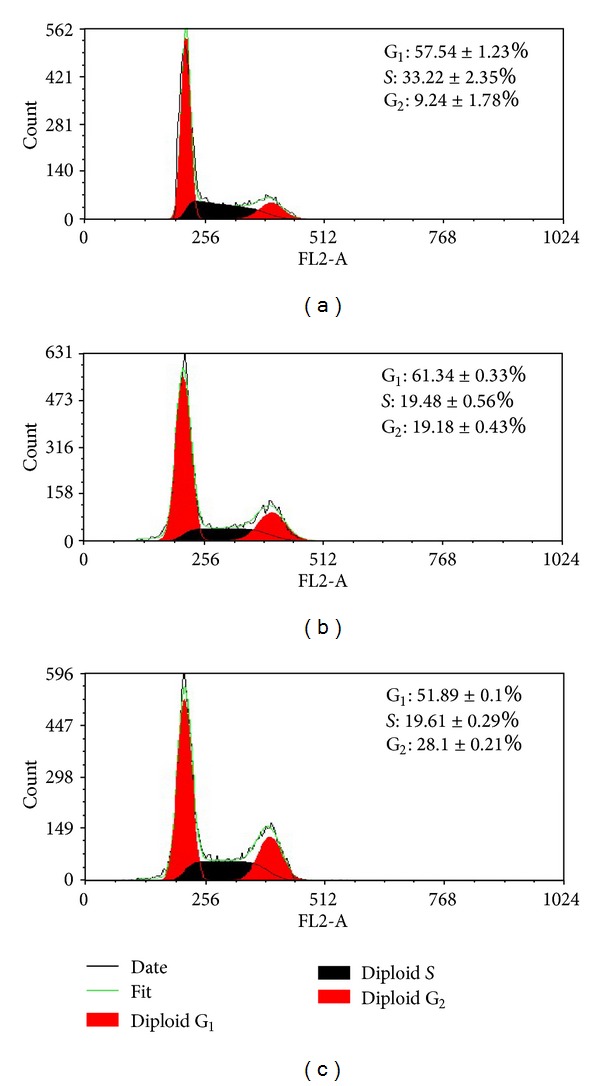
Flow cytometer analysis of cell cycle distribution. Hela cells were treated with free curcumin and the curcumin loaded micelles at the curcumin concentration of 50 *μ*g/mL for 24 h. (a) Control, (b) free curcumin, and (c) the curcumin-loaded 4-arm PEG-b-PAE micelles.

Figure 10 shows the cell cycle histograms of Hela cells treated with blank control, free curcumin, and the curcumin-loaded micelles for 72 h. After the cells were treated with free curcumin or the curcumin-loaded micelles, the percent of cells in G_2_/M phase increased to 19.18 ± 0.43% and 28.10 ± 0.21%, respectively, which differed from blank control (9.24 ± 1.78%). These data suggested that the curcumin-loaded micelles showed more significant cell cycle arrest effect at G_2_/M phase in Hela cells compared to free curcumin at the same concentration. The result appears to be consistent with that of the in vitro cytotoxicity experiments.

### 3.9. Cell Apoptotic Rate Detected by FCM

**Figure 11:**
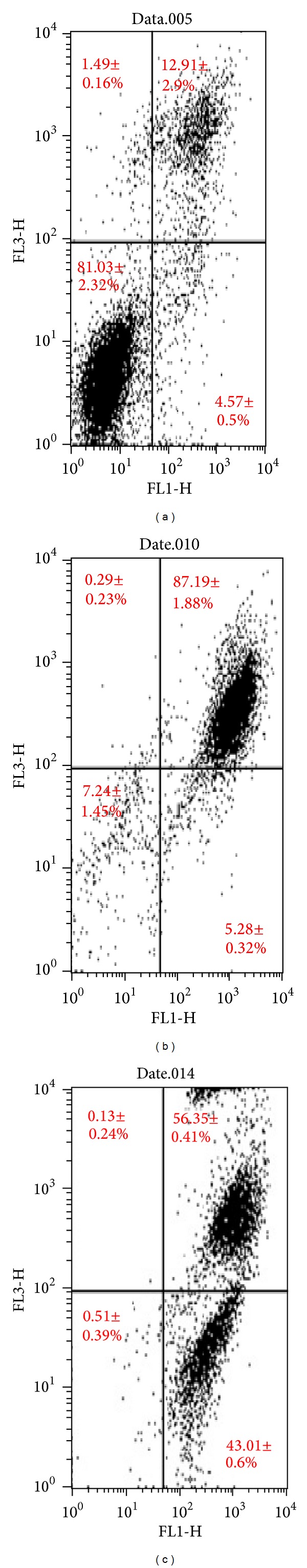
Cell apoptotic rate detected by flow cytometer. Hela cells were treated with curcumin and the curcumin-loaded micelles at the curcumin concentration of 50 *μ*g/mL for 72 h. (a) Blank control, (b) Free curcumin, and (c) the curcumin-loaded 4-arm PEG-b-PAE micelles.

Annexin V/PI double staining is a sensitive method in detecting apoptosis. Externalization of phosphatidyl serine (PS) from the inner side to outer leaflet of the cell membrane is an important indication of early apoptosis. Annexin V possesses a high affinity towards PS; early apoptotic cells can be easily detected by fluorescently labeled Annexin V. Meanwhile, PI can detect necrotic cells due to its permeability through the damaged cell membranes [32]. As shown in Figure 11, after 72 h treatment, the percentages of early and late apoptosis of the curcumin-loaded micelles-treated cells were 43.01 ± 2.60% and 56.35 ± 0.41%, respectively, while those of curcumin-treated cells were 5.28 ± 0.32% and 87.19 ± 1.88%, respectively. The total apoptosis rates of Hela cells caused by curcumin and the curcumin-loaded micelles were 92.47 ± 2.20% and 99.36 ± 0.56%, respectively. Obviously, in comparison with free curcumin, the curcumin-loaded micelles could promote a higher apoptotic rate in Hela cells at the same dose. The result agreed with that of our in vitro cytotoxicity experiments.

## 4. Conclusions

Amphiphilic block copolymer 4-arm poly(ethylene glycol)-b-Poly(anhydride-esters) (4-arm PEG-b-PAE) was successfully synthesized. The 4-arm PEG-b-PAE is able to self-assemble in water to form micelles as curcumin nanocarrier. The critical micelles concentration of the 4-arm PEG-b-PAE was 2.38 *μ*g/mL, which implied the good thermodynamic stability of 4-arm PEG-b-PAE micelles. Curcumin was released from the 4-arm PEG-b-PAE micelles, faster in pH 5.0 than pH 7.4, presenting one biphasic drug release pattern with rapid release at the initial stage and slow release later. The cellular uptake of the the curcumin-loaded micelles in Hela cell increased in a time-dependent manner. The curcumin loaded PAE-b-PEG micelles had higher toxicity to Hela cells than free curcumin, which could induce G_2_/M phase arrest and apoptosis of Hela cells.

## Figures and Tables

**Table 1:** Characterization of the polymers.

Sample	PAE content in copolymer (wt%)^a^	Mn		PDI^b^
		Theoretical	Experimental^a^	
PAE	100		5500	1.58
4-arm PEG-b-PAE	61.09	42000	39010	2.65

^a^Calculated from the^ 1^H-NMR results.

^b^PDI is the polydispersity of copolymers by GPC.

**Table 2:** Characterization of micelles (*n* = 3).

Micelles	Drug loading content (%)	Encapsulation efficiency (%)	Size (nm)	PDI	*ζ*-potential (mv)
4-arm PEG-b-PAE	—	—	147.1 ± 0.1	0.11 ± 0.01	−9.9 ± 1.2
Curcumin/4-arm PEG-b-PAE	7.0 ± 0.2	85.2 ± 1.2	151.9 ± 0.6	0.09 ± 0.05	−10.5 ± 1.3
